# Association between multimorbidity and falls and fear of falling among older adults in eastern China: a cross-sectional study

**DOI:** 10.3389/fpubh.2023.1146899

**Published:** 2023-05-18

**Authors:** Liuqing You, Lihua Guo, Na Li, Jieming Zhong, Yuliang Er, Ming Zhao

**Affiliations:** ^1^Zhejiang Provincial Center for Disease Control and Prevention, Zhejiang, China; ^2^National Center for Chronic and Non-Communicable Disease Control and Prevention, Chinese Center for Disease Control and Prevention, Beijing, China

**Keywords:** multimorbidity, falls, fall risk, fear of falling, older adults, geriatrics

## Abstract

**Background:**

Growing evidence has reported an association between multimorbidity and falls and fear of falling (FOF) in older adults, however, the results regarding this association from China are limited. Our study aimed to investigate the association between multimorbidity and falls and FOF in older adults in eastern China.

**Methods:**

We conducted a cross-sectional study in Zhejiang Province, Eastern China, which recruited a provincial representative sample of adults aged ≥ 60 years. A structured questionnaire including demographic characteristics, chronic diseases, history of falls in the past 12 months, and FOF, was administered by all participants. The exposure variable was multimorbidity, which was defined as the presence of two or more chronic diseases and medical conditions in the same individual. The outcomes included a history of falls and FOF. Multivariate logistic regression was used to evaluate the association between multimorbidity and falls and FOF in older adults.

**Results:**

In total of 7,774 participants were included in the analysis, among whom 3,898 (50.1%) were female, with a mean ± standard deviation age is 72.9 ± 8.4 years. Multimorbidity was associated with the increased risk of falling in older adults [adjusted odds ratio (OR), 1.99; 95% confidence interval (CI):1.55–2.36]. The ORs for having experienced single fall and repeated falls were 1.85 (95% CI: 1.42–2.42) and 3.45 (95% CI: 1.47–6.97), respectively, with multimorbidity compared with those without chronic diseases. The older adults with multimorbidity were more likely to report FOF compared with those without chronic diseases (adjusted OR, 1.49; 95%CI:1.30–1.70). Moreover, the association between multimorbidity and FOF remained significant in the older adults with a history of fall (OR, 1.57; 95%CI:1.04–2.38).

**Conclusion:**

The association between multimorbidity and falls and FOF is significant in the Chinese population and the effects of multimorbidity on falls and FOF do not vary according to the frequency and history of falls in older adults.

## 1. Introduction

Falls are highly prevalent and the second leading cause of unintentional injury deaths worldwide (1). Annually, approximately 37·3 million severe falls occur, leading to a significant loss of >17 million disability-adjusted life-years (DALYs) (1). Older adults are more susceptible to fatal falls and other serious consequences, such as hip fractures (2). According to the Global Burden of Disease Study 2019, the DALYs of falls have been in the top 10 in the 75-years-and-older age groups (3). In China, falls among older adults have also become a major health concern, with the recently estimated incidence and mortality due to falls being 3799.4 and 39.2 per 100,000 populations among individuals aged ≥ 60 years (4).

Progressive studies on falls in older adults are increasingly considering fear of falling (FOF). FOF is defined as a lasting concern with falling that results in restricted activity during an individual’s daily life (5). The occurrence of falls in older adults might contribute to the development of FOF (6), which might, in turn, increase the risk of fall incidents (7, 8), leading to the establishment of a vicious circle. In addition, FOF in older adults may result in other adverse health outcomes such as decreased quality of life (9). FOF is a common issue among older adults, with estimates suggesting that its prevalence among community-dwelling older adults is 20 to 60% (10–15). Given the heavy burden of falls, FOF, and the aging population trend (16), identifying the vulnerable subgroups at high risk of falling and targeting preventive actions toward them have become priorities to prevent and control falls.

Multimorbidity is defined as the co-occurrence of at least two chronic conditions in the same individual (17). It is common among older adults, with a reported prevalence of 65% in those aged 65–84 years (18, 19). Frailty is considered a major threat to multimorbidity in older adults and may lead to physical functional decline and disability (20, 21). Falls and FOF were associated with poor physical function (e.g., balance, gait speed, and grip strength) (22, 23). Therefore, older adults with multimorbidity may be frail and have worse physical function, leading to a higher risk of falls and FOF.

Several epidemiological studies have linked multimorbidity to a higher risk of falls and FOF in older adults (24–26). However, most of these studies have been conducted in developed countries, and evidence from China is limited. Furthermore, there is a study gap regarding whether the effects of multimorbidity on falls or FOF vary depending on the frequency and history of falls among older adults. Therefore, the present study aimed to evaluate the association between multimorbidity and the risk of falls and FOF among the older adults in China and to explore whether there is a difference in the effects of multimorbidity on single vs. repeated falls and whether this association between multimorbidity and FOF still exists in older adults with a history of falls.

## 2. Materials and methods

### 2.1. Study design and participants

The current data analysis was based on a cross-sectional study that used a multistage stratified cluster sampling procedure (Appendix 1) to recruit a representative provincial sample of adults aged ≥ 60 years in Zhejiang Province from June to December 2022. We only included residents living in local communities for >6 months and those who were able to effectively communicate in the sampling frame. Those with critical illness and who could not participate in this survey was excluded. In total of 7,774 participants were included in the analysis. This study was approved by the Ethics Review Committee of the Zhejiang Center for Disease Control and Prevention, and all participants provided written informed consent.

### 2.2. Data collection

We conducted household surveys or centralized surveys to obtain data. Trained interviewers administered a general questionnaire including information on demographic characteristics, fall-related factors, fall occurrence, and injury occurrence. The demographic information included sex, age, region, educational level, marital status, cohabitation, etc. Fall-related factors included illness, medication, physical activity, and the ability to perform activities of daily living of the surveyed participants. The history of falls included the number of falls in the past year, the time, place, behaviors, cause of fall, the direction of fall, on-site disposal, consultation and treatment, and the effect on the life and psychology of the surveyed participants.

### 2.3. Chronic diseases and multimorbidity

Chronic disease was measured according to the participant’s response to the following question: “Have you been diagnosed with any of the following diseases by a physician at a community health center or above?” The diseases included hypertension, diabetes, coronary heart disease, stroke, asthma, chronic bronchitis, arthritis, osteoporosis, osteocalcin, Parkinson’s disease, Alzheimer’s disease, visual impairment, foot disease, vertigo, and tumors. Multimorbidity was defined as having two or more self-reported chronic diseases. The participants were then classified into three groups: none, single, or multiple (two or more).

### 2.4. Falls and fear of falling

In our study, falls were measured according to the participant’s response to the following question: “In the past 12 months, have you ever fallen/stumbled, whether injured or not (yes/no)?” Individuals who had not reported any falls during the period of the last 12 months were classified as a non-faller. Fallers can be classified as single or repeated fallers according to the frequency of falls in the past 12 months. FOF was measured according to the participant’s response to the following question: “At present are you afraid that you may fall over (yes/no)?”

### 2.5. Statistical analyses

The participants were categorized into three groups according to multimorbidity. All descriptive statistics are presented as frequencies and percentages for categorical variables, and as means and standard deviations for continuous variables. Differences were tested using a *t*-test or non-parametric Wilcoxon test for continuous variables and χ^2^ or Fisher’s exact test for categorical variables.

The association between multimorbidity and fall and FOF in the older adults was compared using univariate and multivariate logistic regression analyses. The multivariate model was adjusted for age, sex, region, marital status, educational level, and physical activity. To assess the association between multimorbidity and FOF, falls were included as a covariate. For the number of chronic diseases, a specific regression model with the exposure variables as continuous terms was used. Sensitivity analyses were performed by dividing the disease combinations into three common multimorbidity patterns: cardiopulmonary pattern, musculoskeletal pattern and vascular-metabolic pattern (Appendix 2) based on a previous study (27). To assess whether there was a difference in the effects of multimorbidity on single vs. repeated falls, we conducted a logistics regression with single fall as a control. Because multiple comparisons were involved, we used the Bonferroni correction to reduce the risk of type I errors.

Stratified analyses were performed to identify potential effect modifications by age (years; 60–69, 70–79, and 80–), sex (male, female), region (city, rural), marital status (married, others), educational level (primary school and below, middle, and above), and physical activity (yes, no). To assess the association between multimorbidity and FOF, we also perform a stratified analysis according to the falls (yes/no). *p*-values for the differences were calculated by introducing an interaction term between multimorbidity and the modifier in the regression models. All stratified analyses were adjusted for age, sex, region, marital status, educational level, and physical activity. Fall was included as a covariate in the multimorbidity-FOF analysis.

Statistical analyses were perform using in R software 4.1.0. Two-tailed *p-*values < 0.05 were considered statistically significant. For multiple comparisons, two-tailed *p*-values < 0.167 (Bonferroni correction: 0.05/3, no fall, single fall, repeated fall) were considered statistically significant.

## 3. Results

In total, 7,774 community-dwelling older adults (age, 72.9 ± 8.4 years) participated in this study. 50.1% of the participants were women, 40.5% were aged 60–69 years, 58.3% were residing in rural areas, 76.9% were married, 80.8% had educational level of primary school and below, and 33.3% were engaged in physical activity. The characteristics of the sample are listed in Table 1. The overall prevalence of multimorbidity in the sample was 49.3%, with higher proportions among women (53.5%), those aged 70–79 years (35.8%), those residing in rural areas (55.1%), those married (72.0%), and those with primary school and below (83.0%), and those with no physical activity (67.9%).

**Table 1:** Characteristics of the study population by categories of multimorbidity.

level	All (*n* = 7,774)	None (1,568)	Single (2,376)	Multiple (3,830)	*P*-value
Age (years old)	72.9 ± 8.4	70.3 ± 8.1	72.5 ± 8.3	74.3 ± 8.4	<0.001
60–69	3,152 (40.5)	874 (55.7)	992 (41.8)	1,286 (33.6)	<0.001
70–79	2,611 (33.6)	438 (27.9)	802 (33.8)	1,371 (35.8)	
80 and above	2,011 (25.9)	256 (16.3)	582 (24.5)	1,173 (30.6)	
Sex					<0.001
Male	3,876 (49.9)	844 (53.8)	1,250 (52.6)	1,782 (46.5)	
Female	3,898 (50.1)	724 (46.2)	1,126 (47.4)	2,048 (53.5)	
Region					<0.001
City	3,240 (41.7)	623 (39.7)	898 (37.8)	1,719 (44.9)	
Rural	4,534 (58.3)	945 (60.3)	1,478 (62.2)	2,111 (55.1)	
Marital status					<0.001
Married	5,982 (76.9)	1,323 (84.4)	1,900 (80.0)	2,759 (72.0)	
Widowed	1,633 (21.0)	210 (13.4)	433 (18.2)	990 (25.8)	
Other	159 (2.0)	35 (2.2)	43 (1.8)	81 (2.1)	
Educational level					<0.001
Primary school and below	6,282 (80.8)	1,183 (75.4)	1,920 (80.8)	3,179 (83.0)	
Middle school	1,377 (17.7)	355 (22.6)	423 (17.8)	599 (15.6)	
College and above	115 (1.5)	30 (1.9)	33 (1.4)	52 (1.4)	
Physical activity					0.002
No	5,185 (66.7)	987 (62.9)	1,597 (67.2)	2,601 (67.9)	
Yes	2,589 (33.3)	581 (37.1)	779 (32.8)	1,229 (32.1)	

**Figure 1:**
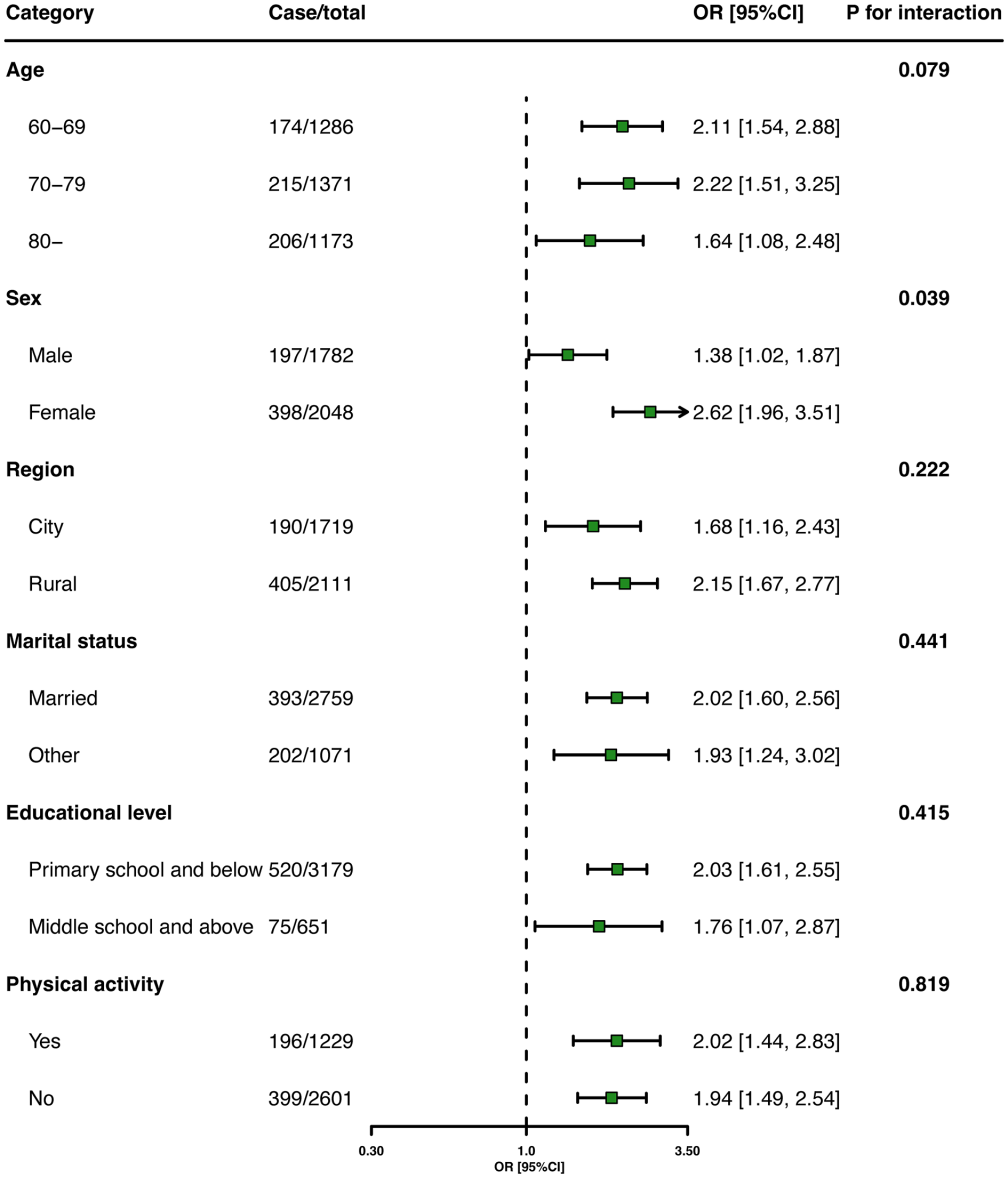
Associations between multimorbidity and falls among older adults in stratified analyses.

Table 2 presents the association between multimorbidity and falls in older adults. The adjusted logistic regression analysis showed that those with multimorbidity were more likely to fall than those without chronic diseases [odds ratio (OR), 1.99; 95% confidence interval (CI), 1.55–2.36]. Stratified analyses showed that the estimated association between multimorbidity and falls persisted among the subgroups (Figure 1).

**Table 2:** Association between multimorbidity and falls among the older adults.

Multimorbidity	No-fall	Fall	Unadjusted model	Adjusted model^*^
None	1,445 (21.1)	123 (13.2)	Ref.	Ref.
Single	2,162 (31.6)	214 (23.0)	1.16 [0.92, 1.47]	1.08 [0.86, 1.37]
Multiple	3,235 (47.3)	595 (63.8)	2.16 [1.76, 2.63]	1.99 [1.55, 2.36]
P for trend			<0.001	<0.001

^*^Adjusted model was adjusted age, sex, area, marital status, education level, physical activity.

Table 3 shows the association between multimorbidity and single and repeated falls in older adults. The ORs of experiencing single and repeated falls were 1.85 (95% CI: 1.42–2.42) and 3.45 (95% CI, 1.47–6.97), respectively, with multimorbidity compared with those without chronic diseases. Case–case analysis showed that there was no significant difference in the effects of multimorbidity on single and repeated falls (OR:1.87, 95%CI:0.87–4.25).

**Table 3:** Association of multimorbidity with single fall and repeated fall among the older adults.

Multimorbidity	No-fall	Single fall		Repeated fall		Repeated fall vs. Single fall
	*N* (%)	*N* (%)	OR [95%CI]^a^	*N* (%)	OR [95%CI]^a^	OR [95%CI]^a^
None	1,445 (21.1)	112 (14.0)	Ref.	11 (8.3)	Ref.	Ref.
Single	2,162 (31.6)	183 (22.9)	1.03 [0.76, 1.39]	31 (23.3)	1.71 [0.73, 4.00]	1.69 [0.69, 4.16]
Multiple	3,235 (47.3)	504 (63.1)	1.85 [1.42, 2.42]	91 (68.4)	3.45 [1.47, 6.97]	1.87 [0.82, 4.25]
P for trend			<0.001		<0.001	

^a^Model was adjusted age, sex, area, marital status, education level, physical activity. Two-tailed *p-*values < 0.167 (Bonferroni correction:0.05/3) were considered statistically significant.

**Figure 2:**
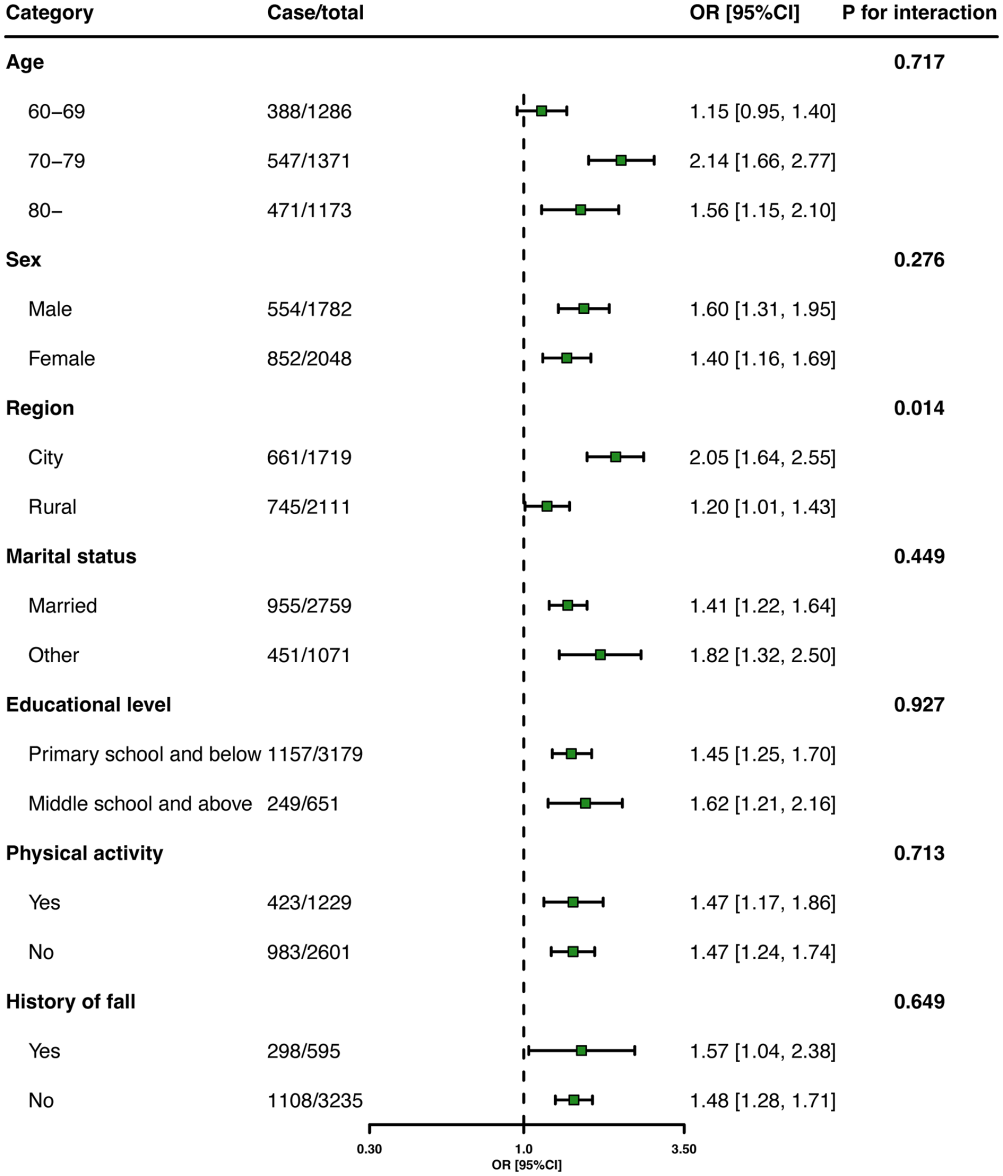
Associations between multimorbidity and FOF among older adults in stratified analyses.

Table 4 shows the association between multimorbidity and FOF in older adults. Adjusted logistic regression analysis showed that older adults with multimorbidity were more likely to report FOF compared with those without chronic diseases (OR, 1.49; 95%CI:1.30–1.70). Figure 2 shows that the estimated association between multimorbidity and FOF among the subgroups. The association between multimorbidity and FOF remained in the older adults with a history of falls (OR, 1.57; 95%CI,1.04–2.38).

**Table 4:** Association between multimorbidity and FOF among the older adults.

Multimorbidity	No-FOF	FOF	Unadjusted model	Adjusted model^*^
None	1,168 (22.0)	400 (16.3)	Ref.	Ref.
Single	1,725 (32.4)	651 (26.5)	1.10 [0.95, 1.27]	1.05 [0.91, 1.22]
Multiple	2,424 (45.6)	1,406 (57.2)	1.69 [1.49, 1.93]	1.49 [1.30, 1.70]
P for trend			<0.001	<0.001

^*^Adjusted model was adjusted age, sex, area, marital status, education level, physical activity, fall.

Sensitivity analysis showed that the association between the three and multimorbidity patterns with falls and FOF remained significant (Appendix Table S1).

## 4. Discussion

The current study explored the association between multimorbidity and falls and FOF in older adults. Older adults with multimorbidity had a significantly higher fall risk compared with those without diseases. In addition, the older adults with multimorbidity were more likely to be afraid of falling when compared with those without chronic diseases. The effects of multimorbidity on FOF did not vary in different subgroups.

Previous studies have indicated a positive association between multimorbidity and falls in older adults. In a Canadian national study comprising 16,357 individuals aged ≥ 65 years, fall risk was significantly greater in individuals with one, two, four, five, and six or more chronic conditions than in those with no chronic conditions (all *p* < 0.05) (25). A population-based cohort study in Sweden demonstrated that individuals defined as “well-functioning with multimorbidity,” had higher risk of falling over a longer follow-up period (5 and 10 years), compared with those in the reference group (5-year hazard ratio [HR] = 1.74 [95% CI, 1.02, 2.66], 10-year HR = 1.44 [95%CI, 1.02–2.04]) (28). A positive association was also observed in a sample of centenarians in which a 38.4% higher OR for a history of falls was associated with the number of health conditions (OR = 1.384 [95% CI 1.087, 1.763]) (29). However, the effect of multimorbidity on single and repeated falls was inconsistent. In a Finland study containing 872 older adults, the number of chronic diseases was only related to the risk of recurrent falling, not to the risk of one-time falling (24).

In our study, we found that the older adults with multimorbidity had a 91% higher odds ratio (OR) for falling than those without a chronic disease. We also classified falls into single (one-time) and repeated (recurrent falls) falls according to the frequency of falls and performed logistic regression with single fall as controls. We found that multimorbidity was significantly associated with the risk of single fall and repeated falls, and that there was no significant difference between the two types of falls. This inconsistency may be due to differences in study setting, the sample size, and the study population. It is very difficult to compare association estimates across studies, however, they all confirmed that multimorbidity was an important risk factor for falls in older adults.

Regarding the mechanism, falls are an inevitable part of the bipedal gait and physical activity (30) and are closely related to physical function such as walking speed and hip strength (31, 32). It has been suggested that multimorbidity often predicts a decline in physical function (33). Moreover, it is associated with poor physical function in older adults (34–36). Hence，multimorbidity may result in physical decline, thereby increasing the risk of falling.

Evidence regarding the association between multimorbidity and FOF is limited. A Brazilian studies demonstrated that the presence of multimorbidity was associated with a higher chance of reporting a FOF (7). Our results were consistent with those of their study and showed that the older adults with multimorbidity were more likely to be afraid of falling than those without multimorbidity. The presence of several chronic diseases can increase negative health self-perception and culminate in frailty (37). Frailty is considered a geriatric syndrome associated with several adverse health outcomes, including the FOF (38). Several studies have reported a robust association between FOF and previous falls (39, 40), and evidence suggests that FOF is not always a consequence of a previous fall (12). In our study, we performed stratified analysis, according to the history of falls, and found that the association between multimorbidity and FOF remained significant in older fallers with previous falls. Our results support the view that FOF is multifactorial and multidimensional.

In addition, older adults with FOF tend to restrict and avoid activities because of a loss of confidence in their ability to perform daily tasks safely (10, 41), which might in turn impact the severity and burden of multimorbidity, leading to the establishment of a vicious circle (42). Hence, attention should be paid to the mental health of older adults with multimorbidity, and psychological support should be provided.

In the future, healthcare professionals should consider assessing multimorbidity as a potential risk factor for falls and FOF in older adults. Identifying and managing chronic conditions in older adults may help prevent falls and improve their quality of life. Public health policies should prioritize the prevention of multimorbidity in older adults to reduce the risk of falls and FOF. This may involve strategies to promote healthy lifestyle behaviors, such as regular physical activity, healthy eating habits, and smoking cessation.

Some limitations of this study should be noted when interpreting the results. First, our study used a cross-sectional design, and we were unable to confirm whether a diagnosis of a certain chronic disease was established before or after the occurrence of falls or FOF. Hence, only an association rather than causality could be inferred between multimorbidity and falls or FOF. Further longitudinal research is required to determine the causal association between multimorbidity and falls or FOF. Second, we used traditional survey methods such as interviews and questionnaires, to capture information about multimorbidity, falls, and FOF from the study participants, which might have been subject to recall or social desirability bias, leading to inaccurate study results. We made every effort to ensure the reliability and accuracy of the data, including pilot testing; training of interviewers; providing clear instructions on how to report multimorbidity, falls, and FOF; and conducting follow-up interviews to confirm the reported incidents. Third, regarding multimorbidity studies, there was no agreement on which diseases should be included in the multimorbidity assessment is missing, making the comparison of study findings difficult. The definition of multimorbidity does not consider the severity or duration of chronic illness, which may influence their risk of falls or FOF. Fourthly, we obtained information about FOF using a single-question inquiry method rather than using the Falls Efficacy Scale-International (FES-I) scale, which might limit the ability to make meaningful comparisons with other studies that have used the FES-I scale. Finally, our sample may only represent the population within one province. All participants in our study were enrolled from Zhejiang Province, which may limit potential confounders, such as ethnicity, race, and social development levels, but restrict the generalizability of our results. Thus, nationwide studies are needed to further confirm these findings.

## 5. Conclusion

The association between multimorbidity and falls and FOF is significant in the Chinese population, and the effects of multimorbidity on falls and FOF do not vary according to the frequency and history of falls in older adults.

## Data availability statement

The original contributions presented in the study are included in the article/Supplementary material, further inquiries can be directed to the corresponding author.

## Ethics statement

The studies involving human participants were reviewed and approved by the Ethics Review Committee of the Zhejiang Center for Disease Control and Prevention. The patients/participants provided their written informed consent to participate in this study.

## Author contributions

LY: conception and design of the study, data analysis and interpretation, and drafting the manuscript. LG: data collection and revision of the manuscript for intellectual content. NL, JZ, and YE: study supervision and revision of the manuscript for intellectual content. MZ: conception and design of the study, data collection, and revision of the manuscript for intellectual content. All authors contributed to the article and approved the submitted version.

## Funding

This work was supported by the Public Interest Technology Application Research Project of Zhejiang Province, grant/award number: LGF22H260018 and the Zhejiang Provincial Federation of Social Sciences, grant/award number: 2022B64.

## Conflict of interest

The authors declare that the research was conducted in the absence of any commercial or financial relationships that could be construed as a potential conflict of interest.

## Publisher’s note

All claims expressed in this article are solely those of the authors and do not necessarily represent those of their affiliated organizations, or those of the publisher, the editors and the reviewers. Any product that may be evaluated in this article, or claim that may be made by its manufacturer, is not guaranteed or endorsed by the publisher.
