# Supplementary material for: Association between multimorbidity and falls and fear of falling among older adults in eastern China: a cross-sectional study
Source: Front Public Health. 2023 May 18;11:1146899. doi: 10.3389/fpubh.2023.1146899 (PMC10234124; doi:10.3389/fpubh.2023.1146899)
Supplement: Supplementary file 1 [file Table_2.DOCX]

**Appendix 1 Detailed description of the sampling method**

A multistage stratified cluster sampling method was used, with specific sampling methods for each stage as follows：

**Stage 1**: 6 municipalities were selected according to geographical distribution and economic development.

**Stage 2**: Within each municipality, one district and one county were randomly selected using the cluster sampling method proportional to the size of the population (PPS method) stratified by urban and rural areas, with the number of elderly people as the auxiliary variable.

**Stage 3**: Within each sampled district, 2 streets were randomly selected using the PPS method; within each sampled county, 2 townships were randomly selected using the PPS method.

**Stage 4**: Within each sampled street, 3 neighborhoods committees were randomly selected using the PPS sampling method; within each sampled township, 3 administrative villages were randomly selected using the PPS sampling method.

**Stage 5**: Within each sampled neighborhoods committee and administrative village, according to the inclusion exclusion criteria, exclude elderly people in known empty households and those who did not meet the criteria, to create a sampling frame of elderly people in each neighborhood committee (administrative village). The elderly was sampled according to gender and age groups using simple random sampling methods; the ratio of men to women was 1:1, and the ratio of the three age groups, 60 to 69, 70 to 79 and 80 and above, was 1.6:1.3:1.

**Appendix 2 Definition of three multimorbidity patterns**

A person with a specific multimorbidity pattern was judged by developing two or more diseases sharing common pathogenic mechanisms. Cardiopulmonary pattern, musculoskeletal pattern and vascular-metabolic pattern were the three common patterns. Cardiopulmonary pattern includes heart disease, asthma, and chronic bronchitis. Musculoskeletal pattern includes arthritis, osteoporosis, and hyperostosis. Vascular-metabolic pattern includes hypertension, diabetes mellitus, stroke, and cancer.

| **Table S1 Association of Multimorbidity pattern with Fall and Fear of falling Among the Elderly** | | | | | | |
| --- | --- | --- | --- | --- | --- | --- |
| Variable | Fall | | | Fear of falling | | |
|  | No | Yes | Adjusted OR (95%CI)^a^ | No | Yes | Adjusted OR (95%CI)^b^ |
| Cardiopulmonary pattern |  |  |  |  |  |  |
| None | 5685 (83.1) | 685 (73.5) | Ref. | 4443 (83.6) | 1927 (78.4) | Ref. |
| Single | 967 (14.1) | 200 (21.5) | 1.62 [1.35, 1.93] | 739 (13.9) | 428 (17.4) | 1.24 [1.08, 1.41] |
| Multiple | 190 (2.8) | 47 (5.0) | 1.88 [1.33, 2.61] | 135 (2.5) | 102 (4.2) | 1.59 [1.21, 2.08] |
| Musculoskeletal pattern |  |  |  |  |  |  |
| None | 5177 (75.7) | 610 (65.5) | Ref. | 4124 (77.6) | 1663 (67.7) | Ref. |
| Single | 1221 (17.8) | 216 (23.2) | 1.52 [1.28, 1.80] | 900 (16.9) | 537 (21.9) | 1.36 [1.20, 1.54] |
| Multiple | 444 (6.5) | 106 (11.4) | 1.96 [1.55, 2.47] | 293 (5.5) | 257 (10.5) | 1.91 [1.59, 2.29] |
| Vascular-metabolic pattern |  |  |  |  |  |  |
| None | 2657 (38.8) | 309 (33.2) | Ref. | 2118 (39.8) | 848 (34.5) | Ref. |
| Single | 3144 (46.0) | 435 (46.7) | 1.11 [0.95, 1.30] | 2398 (45.1) | 1181 (48.1) | 1.15 [1.03, 1.28] |
| Multiple | 1041 (15.2) | 188 (20.2) | 1.50 [1.23, 1.83] | 801 (15.1) | 428 (17.4) | 1.20 [1.04, 1.39] |

Note：^a^Adjusted model was adjusted age, sex, area, marital status, education level, physical activity

^b^Adjusted model was adjusted age, sex, area, marital status, education level, physical activity, fall
